# Impact of Geographic Regions on Overall Survival in Patients With Metastatic Renal Cell Carcinoma: Results From an International Clinical Trials Database

**DOI:** 10.1200/JGO.17.00119

**Published:** 2018-01-11

**Authors:** Andre P. Fay, Rana R. McKay, Xun Lin, Ronit Simantov, Toni K. Choueiri

**Affiliations:** **Andre P. Fay**, Hospital São Lucas/Pontifícia Universidade Católica do Rio Grande do Sul, Porto Alegre, Brazil; **Rana R. McKay**, University of California San Diego, San Diego, CA; **Xun Lin** and **Ronit Simantov**, Pfizer Oncology, New York, NY; and **Toni K. Choueiri**, Dana-Farber Cancer Institute, Boston, MA.

## Abstract

**Purpose:**

Health determinants vary according to geographic region and may affect the outcomes of patients with metastatic renal cell carcinoma (mRCC) treated during clinical trials of targeted therapy. Here, we investigate the overall survival (OS) of patients with mRCC treated in the era of targeted therapy by geographic region.

**Methods:**

We conducted a pooled analysis of patients with mRCC who were treated during phase II or III clinical trials. Clinical characteristics and survival data were collected. Statistical analyses were performed with the Kaplan-Meier method and log-rank test in univariable analysis.

**Results:**

Overall, 4,736 patients were included in the analysis. Patient characteristics differed according to geographic region. No statistically significant differences in OS were observed when the United States/Canada (USC) was compared with the following other regions: Latin America, Asia/Oceania/Africa, and Eastern Europe. In a univariable analysis, OS differed among patients enrolled in trials in USC compared with Western Europe (20.3 *v* 17.4 months; hazard ratio, 1.15; 95% CI, 1.03 to 1.3; *P* = .015), but it did not differ in a multivariable analysis. All-grade treatment-related adverse events (AEs) were observed more frequently in USC. There were no significant differences in grade 3 to 5 AEs among groups.

**Conclusion:**

Despite different baseline characteristics, OS was similar among patients enrolled in clinical trials across different geographic regions. Access to clinical trials as well as disease biology, AE reporting, and quality of care may contribute to potential differences in outcomes.

## INTRODUCTION

Renal cell carcinoma (RCC) is a global health problem.^1^ In 2012, there were more than 300,000 new RCC occurrences, which resulted in approximately 100,000 deaths worldwide.^2^ In addition, the incidence of RCC is increasing worldwide.^3^ Interestingly, mortality rates in developing countries are increasing, whereas mortality from RCC in developed countries has stabilized during recent years.^3^

Breakthroughs in cancer treatment have been achieved in the past few years, which has led to an overall improvement in clinical outcome for patients with metastatic disease. However, major advances in oncology have been restricted to specific populations within and across regions and countries, such as developed counties with increased health care resources.^4,5^ The US Department of Health and Human Services released comprehensive objectives to improve national health outcomes through improved policy and practice.^6^ The 2020 objectives highlight the following message: “To eliminate health disparities and promote health equity, it would be necessary to address all important determinants of health disparities that can be influenced by institutional policies and practices.”^6^ This message has applications globally. Importantly, health determinants—which include differences in personal, social, economic, and environmental factors that determine the health status of individuals or populations—vary according to the geographic regions and country wealth.^7^ The International Agency for Research on Cancer, the specialized cancer agency of WHO, launched the World Cancer Report 2014, a collaboration of more than 250 leading scientists from more than 40 countries, to describe multiple aspects of cancer research and control.^8^ This report highlights the alarming growth rate of the cancer burden and emphasizes the need for implementation of effective strategies to curb this disease globally. Because of growing and aging populations, developing countries are disproportionately affected by the increasing numbers of cancer occurrences.

A better understanding of RCC pathogenesis has resulted in the development of targeted agents that have dramatically changed the natural history of patients with advanced disease.^9,10^ Clinical trials in metastatic RCC (mRCC) to test different targeted agents have been conducted worldwide; have enrolled patients from different geographic regions, such as the United States, Canada, Europe, Latin America, Asia, and Africa; and have led to the approval of several agents that are now widely used as the standard of care in the metastatic setting.^11,12^ However, the impact of geographic region, country income status, and country life expectancy on the clinical outcomes of patients with mRCC treated with targeted therapy has not been explored.

Health determinants vary according to geographic region and may impact the outcomes of patients with mRCC treated during clinical trials of targeted therapy. Increased knowledge about population diversities and health determinants provides a unique opportunity to improve barriers in cancer care across geographic regions and countries and to understand mechanisms of genomic diversity that may affect the clinical outcome of patients with mRCC.^1^ Given the lack of data about efficacy and safety of targeted therapies in patients with mRCC according to the geographic region, we sought to investigate the impacts of the geographic region, country income, and country life expectancy on clinical outcomes of patients with mRCC who are treated in the era of targeted therapy.

## METHODS

### Study Design

We conducted a pooled retrospective analysis of patients with mRCC treated during phase II (Clinicaltrials.gov identifiers: NCT00054886, NCT00077974, NCT00267748, NCT00338884, NCT00137423, and NCT00835978) and phase III Clinicaltrials.gov identifiers: NCT00083889, NCT00065468, NCT00678392, NCT00474786, NCT00631371, and NCT00920816) clinical trials sponsored by Pfizer. The database included 4,736 patients with mRCC treated between 2003 and 2013.

Baseline demographic characteristics, clinicopathologic features, and survival and toxicity data were collected across the following geographic regions: United States/Canada (USC), Western Europe (WE), Eastern Europe (EE), Latin America (LA), and Asia/Africa/Oceania (AAO). In addition, patients were categorized by country income status, which was defined as high, upper-middle, and lower-middle according to data from the World Bank,^13^ and by country-specific adult life expectancy with data from a 2015 WHO report^14^ (Appendix Table A1). For country-specific life expectancy, countries were dichotomized into two groups—high and low—on the basis of the median of the adult life expectancies of countries in our cohort.

### Study End Points

The primary end point of this study was to evaluate overall survival (OS) by the geographic region. OS was defined as the time from initiation of therapy to death as a result of any cause, censored at last follow-up. Secondary end points included evaluation of OS by country income status and country-specific adult life expectancy as well as assessments of progression-free survival (PFS), objective response rate (ORR), and safety by geographic region, country income status, and country-specific adult life expectancy. PFS was defined as the time from initiation of therapy to date of progression or death as a result of any cause or was censored at last follow-up. ORR was defined by RECIST version 1.1. Treatment-associated toxicities were evaluated with the Common Terminology Criteria for Adverse Events, version 3.0, and any-grade and grade 3 to 5 toxicities were described.

### Statistical Analyses

OS and PFS were estimated with the Kaplan-Meier method. Associations between OS and PFS were assessed by using the log-rank test in univariable analysis, and multivariable Cox regression analysis adjusted for significant variables in the univariable analysis. USC was used as a reference for comparisons of geographic region. High income was used as a reference for country income comparisons. High country-specific life expectancy was used as the reference for life expectancy comparisons.

All statistical analyses were performed with SAS version 9.2 (SAS Institute, Cary, NC). A two-sided *P* value of < .05 was considered statistically significant.

## RESULTS

### Baseline Characteristics

Overall, 4,736 patients from different geographic regions were included in the analysis: USC (n = 1,544; 32.6% of total), AAO (n = 1,254; 26.47%), WE (n = 897; 18.94%), EE (n = 792; 16.72%), and LA (n = 250; 5.27%). Patient characteristics differed by geographic region; they are listed in Table 1. Briefly, patients in USC and WE were slightly older (mean ages, 60.6 and 60.5 years, respectively). An Eastern Cooperative Oncology Group (ECOG) performance status (PS) of 0 was more frequent in patients in LA. Body mass index (BMI) differed across regions (range of BMI > 25 kg/m^2^, 44% to 80%); higher BMI was observed more often in USC and LA. In addition, the number of patients who underwent a prior nephrectomy was higher in USC and WE. Baseline hypertension was more frequent in USC. There were more poor-risk patients in EE. In addition, statin and angiotensin system inhibitor (ASI) use was more frequent in USC (Table 1).

**Table 1:**
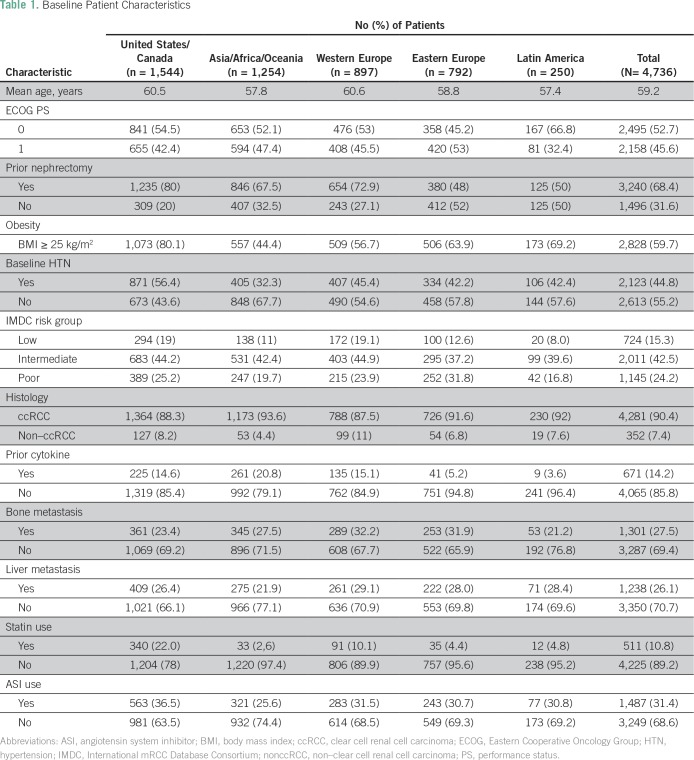
Baseline Patient Characteristics

The numbers of patients enrolled in high-income, upper-middle–income, and lower-middle–income countries were 3,967 (83.76%), 502 (10.6%), and 267 (5.64%), respectively. For country-specific adult life expectancy, 3,218 (68%) patients were categorized into countries with lower than the median adult life expectancy.

### Survival Outcomes

**Fig 1:**
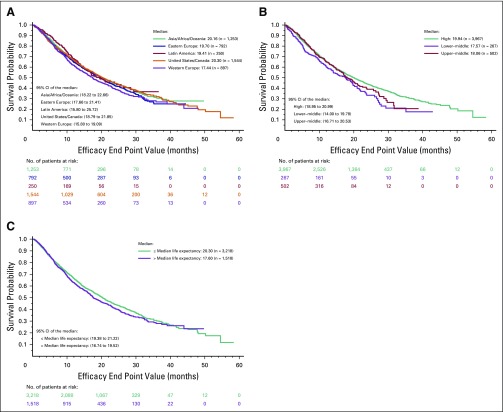
Kaplan-Meier plots of overall survival by (A) geographic region, (B) income, and (C) median life expectancy.

**Fig 2:**
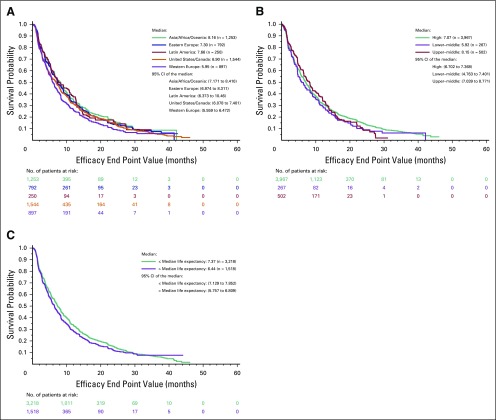
Kaplan-Meier plots of progression-free survival by (A) geographic region, (B) income, and (C) median life expectancy.

The median OS times were 20.3 months in USC, 20.2 months in AAA, 19.7 months in EE, 19.4 months in LA, and 17.4 months in WE (Fig 1A). In a univariable analysis, no statistically significant differences in OS were observed when USC was compared with other regions, specifically wit LA, AAO, and EE. However, OS differed significantly among patients enrolled in trials in WE compared with USC (17.4 *v* 20.3 months; hazard ratio [HR], 1.15; 95% CI, 1.03 to 1.3; *P =* .015; Table 2). In the multivariable analysis, which included only significant variables from the univariable analysis, this difference was no longer statistically significant (HR, 1.12; 95% CI, 0.98 to 1.27; *P* = .08). In decreasing order, the median PFS times were 8.2 months in AAO, 7.6 in LA, 7.3 months in EE, 6.9 months in USC, and 5.9 months in WE (Fig 2A). However, there were no statistically significant differences in PFS by geographic region (Table 2).

**Table 2:**
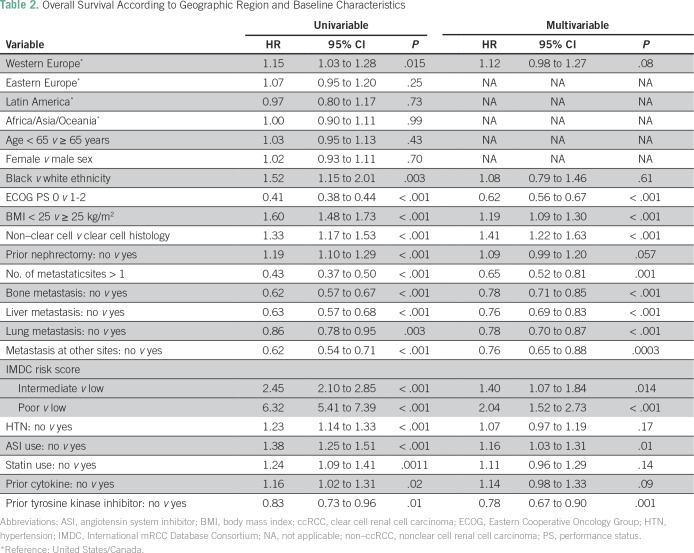
Overall Survival According to Geographic Region and Baseline Characteristics

Median OS times, when stratified by country income, were 19.9 months for high-income countries, 18.0 months for upper-middle–income countries, and 17.5 months for lower-middle–incomes. There were no statistically significant differences in OS (Fig 1B). Similar trends were observed for PFS when stratified by country income (Fig 2B).

Median OS times, when stratified by country-specific median adult life expectancy, were 20.3 months for higher life expectancy and 17.6 months for lower life expectancy (Fig 1C). Again, there were no statistically significant differences in OS. Similar trends were observed for PFS (Fig 2C).

### Response Rates

The ORR for the overall cohort was 24.5%. ORR was similar by geographic region (range, 17.3% to 28.8%), country income (range, 21.4% to 25.1%), and country-specific adult life expectancy (20.4% to 26.4%; Table 3).

**Table 3:**
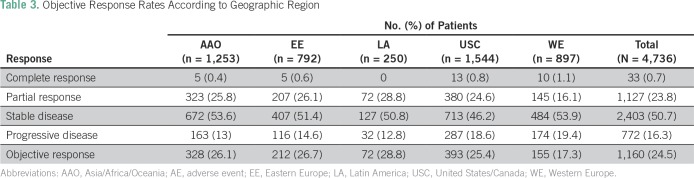
Objective Response Rates According to Geographic Region

### Treatment Exposure and Toxicities

Overall, 1,484 patients (31.3%) underwent a dose reduction or modification: 21.3% of patients in AAO; 28.9%, in EE; 24%, in LA; 41.1%, in USC; and 32.7%, in WE. Reasons for treatment discontinuation included toxicity (n = 675; 14.3%), disease progression (n = 2,017; 42.5% %), and other reasons (n = 2,044; 43.2%).

Overall, the most frequent any-grade treatment-related AEs were diarrhea (n = 2,079; 43.9%), fatigue (n = 2,018; 42.6%), nausea (n = 1,603; 33.8%), decreased appetite (n = 1,580; 33.4%), hypertension (n = 1,272; 26.8%), vomiting (n = 1,091; 23%), weight decrease (n = 1,058; 22.3%), rash (n = 1,046; 22%), pyrexia (n = 1,026; 21.6%), and palmar-plantar erythrodysesthesia syndrome (PPES; n = 1,022; 21.6%; Table 4). Overall, any-grade treatment-related AEs were more prevalent in USC.

**Table 4:**
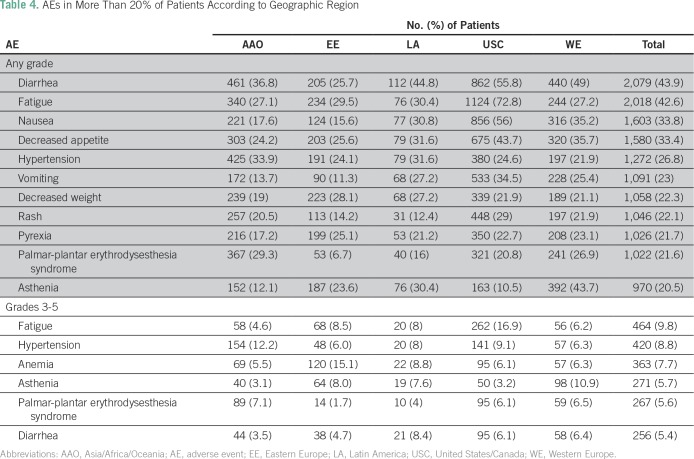
AEs in More Than 20% of Patients According to Geographic Region

The incidence of treatment-related grade 3 to 5 toxicities was low in overall cohort (Table 4). Grade 3 to 5 hypertension was most frequent in AAO (12.2%), followed by USC (9.1%). Lower rates of grade 3 to 5 hypertension were observed in EE (6.0%). Grade 3 to 5 diarrhea was observed in 8.4% of patients in LA, which was higher than the frequency observed in other regions. Also, grade 3 to 5 PPES was reported in 7.1% of patients in AAO, in 6.5% of patients in WE, and in 6.1% of patients in USC. Overall, there were no significant differences in grade 3 to 5 AEs according to geographic region (Table 4).

## DISCUSSION

Cancer care delivery varies significantly across the different global regions. Factors that influence cancer care delivery include system resources and allocation of resources across levels of care, quality of care, finances, and cultural barriers. These variables have the potential to affect survival outcomes for patients.^15^ In this study, we evaluated the survival outcomes of patients with mRCC treated in different global geographic regions. To our knowledge, this is one of the largest analyses to evaluate the impact of geographic region, country income status, and life expectancy on clinical outcomes of patients with mRCC treated in the era of targeted therapy and as part of clinical trials. An understanding of the impact of health determinants in different populations may help eliminate health disparities and promote health equity in cancer treatment.^16^

Our analysis highlights the unique clinical and disease characteristics of patients enrolled in clinical trials in different geographic regions. In our analysis, patients in WE and USC were older than those in other regions. In addition, the majority of patients in LA had an ECOG PS of 0 and 32.4% had an ECOG PS of 1. In EE, AAO, WE, and USC, the rates of ECOG PS of 1 were 53%, 47.4%, 45.4%, and 42.2%, respectively. A potential explanation for the age differences across regions could be explained in part by heterogeneity in access to care across age groups. Also, cultural attitudes and preference for traditional versus modern therapies may explain the age differences observed in our cohort. There were some differences in International Metastatic mRCC Database Consortium prognostic score^17^ distribution by region. More patients with unfavorable-risk disease were reported in EE, and more with favorable-risk disease were reported in USC and WE. Although variations in underlying disease biology may explain these differences, access to care also may be a contributing factor. Recognition of illness and of the potential benefits of treatment is a prerequisite for health care demand. In areas where a larger proportion of the population is in poor health, illness may not be as easily recognized; therefore, patients may present with more overwhelming disease. In addition, increased use of radiographic imaging modalities in developed countries may allow detection of disease at earlier stages. Prior nephrectomies were performed more frequently in USC (in 79.9% of patients). Similar patterns of surgical treatment of primary tumors were observed in WE (72.9% of patients). Interestingly, only 47.9% of patients in EE and 50% in LA had nephrectomy before enrollment in clinical trials. These findings highlight the varied access to surgical treatment may across different regions. Our analysis also revealed different patterns in terms of cytokine use. Prior use of cytokines was frequent in AAO (20.8%), but use was limited in LA and EE. Prior cytokine use was reported in 14.5% of patients in USC and in 15.5% in WE. This difference may reflect differences in access to health care and targeted therapies. Interestingly, similarities in rates of liver or bone metastasis were observed in different regions, which suggests similar aspects of disease biology and pathogenesis.

Moreover, many cancer types are considered metabolic diseases. However, the contribution of metabolic dysfunction to the onset of cancer development remains poorly understood.^18^ Obesity is a well-established risk factor for kidney cancer^19^ and may affect the clinical outcome of patients with advanced disease.^20^ It is predicted that 50% of men and 60% of women in Latin America will be obese by 2030.^21^ Conversely, approximately, 26% of adults are obese in a high-income country like the United States, and approximately 40% are not physically active. In Brazil, a middle-income country, 17% of adults are obese, and a lower number of people are not physically active (14.9%). In our cohort, a BMI of 25 kg/m^2^ or greater was observed in 70.1% of patients in USC and in 69.2% of patients in LA. Interestingly, obesity was less frequent in EE, WE, and AAO (63.8%, 56.7%, and 44.4%, respectively). Societies that experience transitions in nutrition are at the greatest risk of dealing with the burden of simultaneous over- and under-nutrition and cancers associated with each domain. In addition, recent publications have suggested an association of statin and ASI use with improved survival in patients with mRCC treated with targeted therapy.^22^ In our cohort, ASI and statin use differed across different regions and was more prevalent in USC. This may be related to the older more obese patient population, with more comorbidities, in USC. It also may reflect increased access to and increased thresholds for pharmacologically treated hypertension and dyslipidemia.

Recently, Goss et al^15^ highlighted the growing cancer threat in LA and suggested that, with rapidly increasing incidence and mortality rates for cancer, the degree of human suffering and economic burden of cancer will have a negative impact on the clinical outcome of patients with cancer.^15^ Similarly, China, India, and Russia also share a rapidly increasing cancer incidence and mortality that are nearly twice as high as those in Europe or the United States.^23^ The CONCORD-2 study, designed to perform a long-term worldwide surveillance of cancer, has shown important disparities in cancer mortality in different countries. In this registry, survival rates were observed across 67 countries. As an example, the 5-year survival range for patients diagnosed with colon cancer was 50% to 59% in many countries. However, in North America, Oceania, some European countries, and a few countries in Central and South America and Asia, the 5-year survival reached 60% or more. Interestingly, the rate was less than 40% in India, Indonesia, and Mongolia.^24^ RCC survival was not included in this analysis. In our analysis, despite different baseline characteristics, OS, PFS, and ORR were similar among most geographic regions. It is important to note that patients included in our analysis are part of clinical trials, and their clinical outcomes may not reflect the real-world population. Clinical studies provide an opportunity to give patients access to health care and drugs that otherwise will not be available. We hypothesize that, if we are able to provide patients access to standard and experimental treatment approaches, clinical outcomes might not be so divergent across geographic regions. Interestingly, a statistically significantly shorter OS was observed in WE compared with USC in a univariable analysis (20.3 *v* 17.4 months). However, the significance was not maintained in a multivariable analysis, which suggests that outcomes may not be so divergent if patients have access to care or clinical trials. We also did not identify differences in clinical outcome according to country income or life expectancy.

Recently, studies have demonstrated that higher rates of toxicity related to cancer treatment may occur more frequently in Asian patients than in white populations.^25^ In our analysis, we did not identify significant differences in any-grade or grade 3 to 5 AEs across different geographic regions. However, AEs were observed more frequently in USC. In addition, though a broad range of targeted therapies was included in our analysis, not every region enrolled patients in all the clinical trials, and data about recently approved agents, such as cabozantinib and nivolumab, are lacking.

Cancer is a genomic disorder, and its development depends on the interaction of genetic and environmental factors that result in different disease phenotypes.^26^ Geographic differences in cancer incidence, prognosis, and clinical outcome may be related to genomic diversity.^27^ Recently, the molecular characterization of RCC was reported, and genomic alterations have been associated with clinical outcomes and response to treatment.^28,29^ Interestingly, not all populations have been represented in these studies, and the RCC biology within each distinct ethnicity has not been characterized. It is important to note that many widely used genomic platforms available in western countries are not yet available in developing countries. Therefore, the characterization of populations across different regions may improve the understanding of the biology of RCC in different populations and guide rational global investments. As observed in prostate cancer, ethnicity may play a role in the clinical course of RCC. African Americans have higher incidences of prostate cancer and a higher mortality rate than white patients.^30^ Recent studies have suggested that the *TMPRSS2*-*ERG* gene fusion is significantly different in white, African American, and Japanese populations.^31^ These findings have opened avenues to understand disparities observed in different populations. Whether a biologic basis exists to justify differences in mRCC clinical outcome across geographic regions remains unclear and still must be explored.

We evaluated a large cohort of patients with mRCC by using prospectively collected clinical trials data, but our study has many limitations. First, enrollment of all patients in clinical trials, limits the generalizability of our data. In addition, though a broad range of targeted therapies was included in our analysis, not every region enrolled patients in all the clinical trials in our cohort, so data about recently approved agents, such as cabozantinib and nivolumab, are lacking. Finally, lack of information about biology from the different population may affect the results of this analysis.

During the past decade, there has been a paradigm shift in the treatment of cancer driven by advances in personalized medicine and immuno-oncology. Access to improved therapeutic options can change the outcomes of patients globally affected by mRCC. In our cohort, we observed differences in patient and treatment characteristics according to the geographic regions, and these differences may play a role in the reported efficacy and safety of targeted therapies. A better understanding of factors that may contribute to these differences—including different disease biology, access to care, data reporting, and quality of care—must be explored to better inform attempts at personalized medicine across the globe. We highlight that, despite different baseline characteristics, OS was similar among patients enrolled in clinical trials across different geographic regions. Access to clinical trials may be an important alternative to eliminate health disparities and promote health equity in patients with mRCC.

## Figures and Tables

**Table A1:**
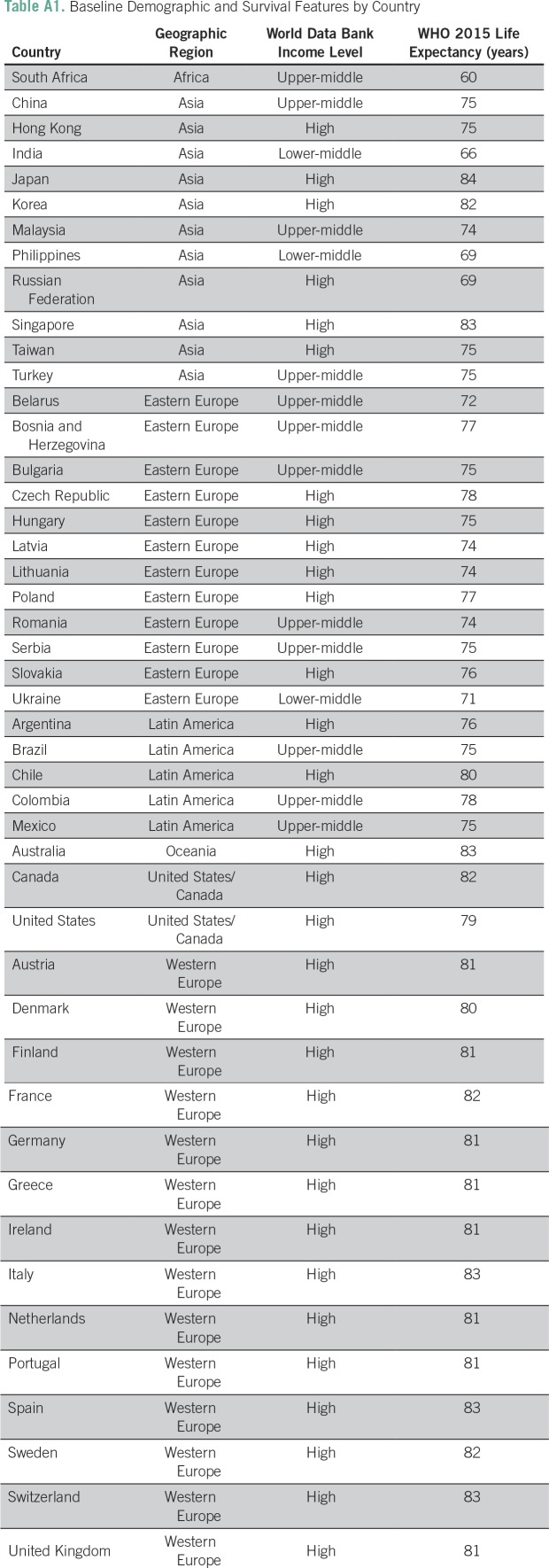
Baseline Demographic and Survival Features by Country
